# Bismuth Oxysulfide and Its Polymer Nanocomposites for Efficient Purification

**DOI:** 10.3390/ma11030447

**Published:** 2018-03-19

**Authors:** Yidong Luo, Lina Qiao, Huanchun Wang, Shun Lan, Yang Shen, Yuanhua Lin, Cewen Nan

**Affiliations:** State Key Laboratory of New Ceramics and Fine Processing, School of Materials Science and Engineering, Tsinghua University, Beijing 100084, China; ydluozd@163.com (Y.L.); qln13@mails.tsinghua.edu.cn (L.Q.); wanghc12@mails.tsinghua.edu.cn (H.W.); lans13@mails.tsinghua.edu.cn (S.L.); shyang_mse@mail.tsinghua.edu.cn (Y.S.); cwnan@mail.tsinghua.edu.cn (C.N.)

**Keywords:** bismuth oxyselenide, high-efficiency purification, defects

## Abstract

The danger of toxic organic pollutants in both aquatic and air environments calls for high-efficiency purification material. Herein, layered bismuth copper oxychalcogenides, BiCuSO, nanosheets of high photocatalytic activity were introduced to the PVDF (Polyvinylidene Fluoride). The fibrous membranes provide an easy, efficient, and recyclable way to purify organic pollutant. The physical and photophysical properties of the BiCuSO and its polymer composite were characterized by scanning electron microscopy (SEM), X-ray diffraction (XRD), ultraviolet-visible diffuse reflection spectroscopy (DRS), X-ray photoelectron spectroscopy (XPS), electron spin resonance (EPR). Photocatalysis of Congo Red reveals that the BiCuSO/PVDF shows a superior photocatalytic activity of a 55% degradation rate in 70 min at visible light. The high photocatalytic activity is attributed to the exposed active {101} facets and the triple vacant associates $V_{Bi}^{\prime \prime \prime }V_{O}^{••}V_{Bi}^{\prime \prime \prime }$. By engineering the intrinsic defects on the surface of bismuth oxysulfide, high solar-driven photocatalytic activity can be approached. The successful fabrication of the bismuth oxysulfide and its polymer nanocomposites provides an easy and general approach for high-performance purification materials for various applications.

## 1. Introduction

Human agricultural and industrial activities can release environment pollutants into both aquatic and air environments [1,2]. Photocatalysts, which can directly convert solar energy into chemical energy, representing a relatively simple and environmentally-friendly route for environmental remediation issues, have been widely studied in the past decades [3,4]. TiO_2_ is one of the most interesting photocatalysts considering its low cost, high stability, environmental benefits, and its potential in commercial applications. The TiO_2_-based polymer composites are also fully studied [5,6]. The polymer coating not only act as the carrier but also the modifier.

Recently, the development of electrospun filter materials has attracted much attention for its advantage of possessing ultrathin diameters (10–1000 nm), scalability of fabrication methods using different source materials, and capability for fabricating polymer composites [7,8,9,10,11]. The effects of decreased fiber diameter on the reduction of air resistance have been fully studied [12,13,14]. Moreover, composite polymer nanofibers containing nanoparticles, which enhance the contaminant capture capability due to fortissimo electrostatic force of nanofibers have been reported [15].

However, increased efficiencies of capturing and degrading contaminants is usually achieved at the cost of the structural imperfections caused by high loadings of ceramic nanoparticles [16,17]. With higher loading, the larger diameter of the nanofibers will results in decreased air permeability [18]. Choosing the ceramic nanoparticles doped in the polymer that form more polar bonds with the polymer matrix can enhance the electric potential differences between the nanoparticles and the polymer and therefore promote the contaminant capture capability [19].

The instinctive shortcoming of TiO_2_-based photo-catalysts is that they are only capable of utilizing the ultraviolet light (less than 5% of the solar light) and/or a very small quantities of visible light due to their wide band gaps, which greatly limit their photocatalytic performance. Numerous attempts have been made to explore ways to utilize visible light, including metal or nonmetal elements doping to narrow the band gap, and heterostructures designed with other photoresponsive materials, for example, carbon-based materials and metallic sulfide. However, photocatalysts with appropriate band gaps that can directly utilize solar light still need to be developed.

Bismuth oxysulfide have attracted great interest in the typical layered structure of bismuth oxysulfide with the stacking of [Bi_2_O_2_]^2+^ layers along with z axis enabling the possibility of the formation of ultrathin nanosheets which may have larger active surface to form more polar bonds with the polymer matrix. In addition, the lone-pair distortion of the Bi 6s orbital in these semiconductors may cause pronounced overlap of O 2p and Bi 6s orbitals in the valence band, which benefits the mobility of photogenerated charge carriers, resulting in improvements of photocatalytic activity comparable to, or even better than, that of anatase TiO_2_ [20,21]. In particular, recent works indicate that the bismuth-based oxides exposed with {001} facets exhibit excellent photoactivity [22,23]. Our previous work demonstrated that new Bi-based layered structured BiCuSeO oxyselenides possess good photocatalytic activity and chemical stability in the full solar light spectrum for degradation of organic contaminants (Congo Red, the most widely used azo dye in textile industry, is used as a model pollutant) in aqueous solution. Thus, loading with oxychalcogenide in the polymer matrix may be a good choice for increasing efficiencies of capturing and degrade contaminant [17].

Herein, pure BiCuSO and vacant BiCuSO were synthesized easily via a hydrothermal method with temperatures of 200 °C and 120 °C, then the vacant BiCuSO nanoplates were coated by PVDF through ESP (electrospinning) method and the photocatalytic activity was investigated. The bismuth oxysulfide BiCuSO and its polymer nanocomposites exhibited remarkable photocatalysis. The highest activity of the PVDF/vacant BiCuSO (hereinafter to be referred as PVDF/vacant BiCuSO) shows a 55% degradation rate in 70 min at visible light. The mechanism of the highly photocatalytic activity is also studied. By taking the narrow band gap ceramic nanoparticle as the filler in the polymer matrix, the resultant composites show potential applications in recyclable filters with visible-light-cleanable features.

## 2. Results

### 2.1. Morphological and Structural Characterization

**Figure 1:**
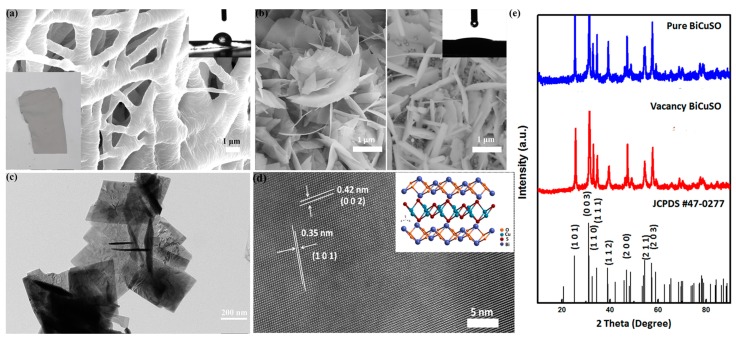
(**a**) SEM patterns of the PVDF/BiCuSO nanofibers; Inset: Left—Digital photograph of PVDF/BiCuSO nanofibers. Right—Side-view images of a water drop on PVDF/BiCuSO film. The contact angles were measured with a DataPhysics goniometer setup. (**b**) SEM patterns of the as-prepared samples with temperature of Left—120 °C B1; Right—200 °C B2; Inset: Side-view images of a water drop on BiCuSO bulk. (**c**,**d**) TEM images of plate-like microcrystals (sample B1); and the corresponding HRTEM image. (**e**) XRD patterns of the as-prepared samples with temperature of 200 °C and 120 °C (B1 and B2).

The morphology of as-prepared samples was characterized by scanning electron microscopy (SEM) and transmission electron microscopy (TEM) as shown in Figure 1. As shown in Figure 1a the diameter of PVDF/BiCuSO was ~3 μm. The composite nanofibers showed a rough surface. The Figure 1b showed the morphologies of vacant BiCuSO and pure BiCuSO. Compared with pure BiCuSO, the vacant BiCuSO nanosheets were generally thinner. The Brunauer–Emmett–Teller (BET) analysis demonstrated that the specific surface areas of crushed powder samples of them are 31.64 m^2^/g and 26.40 m^2^/g respectively as shown in Table 1.

**Figure 2:**
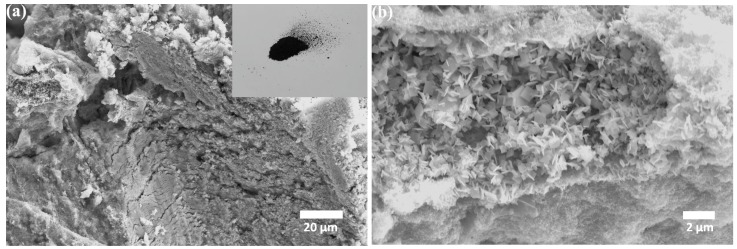
SEM patterns of vacant BiCuSO at various magnifications. Inset: A digital photograph of vacant BiCuSO powders.

The polycrystalline nature of the as-prepared BiCuSO nanosheets was characterized by transmission electron microscopy. As revealed in Figure 1c, the plate-like microcrystals of pure BiCuSO were overlapping irregularly. The corresponding high-resolution transmission electron microscopy (HRTEM) image shows the spacing of some lattice fringes is 0.35 nm, which is close to that (0.352 nm) of the (101) crystal plane of BiCuSO (JCPDS #47-0277). The X-ray diffraction (XRD) shown in Figure 1e reveals that all diffraction peaks can be well indexed to the pure phase of BiCuSO (JCPDS no. 47-0277), without impurity peaks appearing in all as-prepared samples. As the reaction temperature decreases from 200 °C to 120 °C, the relative intensity of the (111) reflex decreases as compared with the (110) reflex. Figure 2 are the SEM patterns of vacant BiCuSO at various magnifications. The plate-like crystallites formed stacks. The changes in relative intensities may attribute to a texture effect induced by the non-statistical orientation of the crystallites and the formation of the vacancy.

### 2.2. Photocatalytic Activities

**Figure 3:**
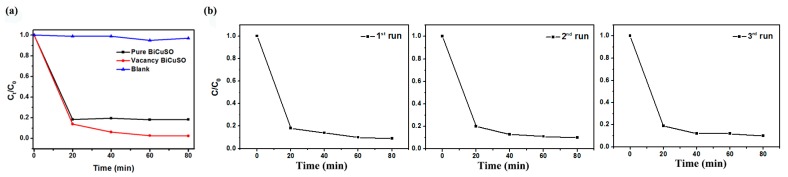
(**a**) Degradation rate of Congo Red aqueous solution with pure BiCuSO and vacant BiCuSO as catalysts under visible light over 80 min; (**b**) cycling runs using BiCuSO powders under light irradiation.

The photocatalytic activities of pure BiCuSO and vacant BiCuSO were evaluated by monitoring the decomposition of the model pollutants Congo Red (CR) aqueous solution under visible light. As shown in Figure 3a, the vacant BiCuSO exhibited strongly enhanced photocatalytic activity compared to pure BiCuSO. The photodegradation rate of Congo Red reached nearly 90% in 20 min and 99% in 1 h. The photocatalytic process basically involves adsorption–degradation–releasing process, and the adsorbed organic pollutions on the surface of photocatalysts is a prerequisite for a photo-induced reaction. For a clear quantitative comparison, we applied the Langmuir−Hinshelwood model, which is designed for photodegradation with the concentration of organic pollutants in the millimolar range, as expressed by

(1) $$\mathrm{In}(C_{0}/C_{t})=kt$$

where *C*_0_ and *C_t_* are the concentrations of pollutant in a solution at times *t*_0_ and *t*, respectively, and *k* is the kinetic constant calculated from the expression [24]. The kinetic constant for the BiCuSO and vacancy BiCuSO under visible light are 0.003 min^−1^ and 0.035 min^−1^ after 20 min, respectively. The above results confirm that the vacant BiCuSO exhibits a higher photocatalytic activity than that of pure BiCuSO. Generally, three processes are involved in photocatalysis, i.e., (i) illumination inducing a transition of electrons from valence band (VB) to conduction band (CB), leaving holes at the top of VB; (ii) transport and recombination of electron-hole pairs; and (iii) redox reaction on the surface of photocatalyst. Since BET analysis demonstrated that the specific surface areas of the nano-sheets samples are 26.40 m^2^/g and 31.64 m^2^/g respectively, indicating negligible influence of the surface area on the photocatalytic performance. Therefore, the generation and transport properties of the photogenerated electron–hole pairs should be responsible for the improvement of photocatalytic property with the introduction of vacancy. The apparent reaction rate constant per unit of surface area (*K*) was calculated to get deeper insight over the effect of hybrid structure according to the formula

(2) $$K=\frac{k}{m\cdot S}$$

where *k* is the apparent reaction rate constant, m is mass of photocatalyst used, and *S* is the specific surface area. Constant *K* implies the degradation rate on unit surface area. As shown in Table 2, we can clearly found that the degradation rates were enhanced in vacant BiCuSO. The reusability of the BiCuSO was determined for the cost effectiveness of the process. The BiCuSO was filtered after photo catalytic degradation, washed several times with water and ethanol, and dried. The dried BiCuSO photocatalyst was used for the degradation of Congo Red molecules under similar experimental conditions. As shown in Figure 3b, the regenerated BiCuSO was effectively used for the degradation up to three cycles under light irradiations. The results proved that the BiCuSO acts as an effective photo catalyst for Congo Red.

**Figure 4:**
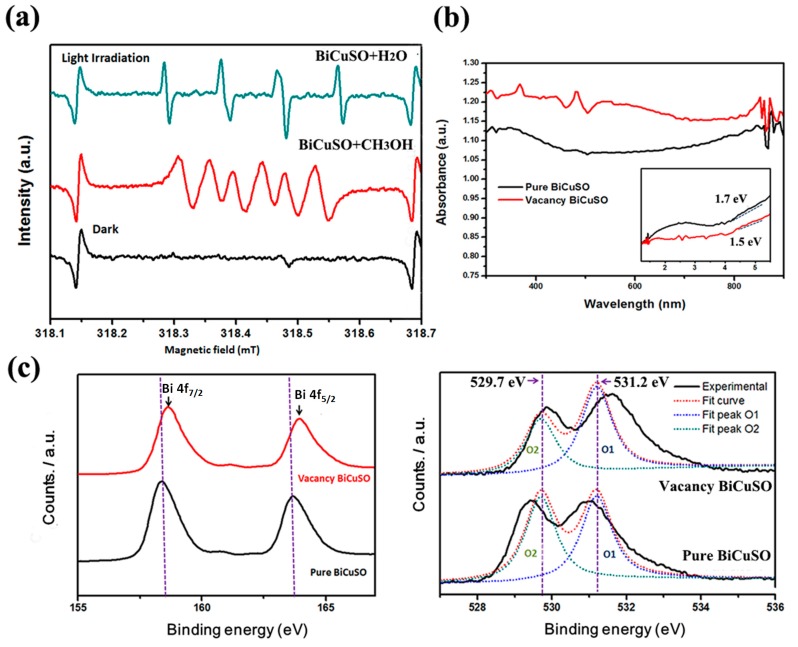
(**a**) ESR signals of the DMPO–trapped •OH (Green) and DMPO-trapped O_2_•^−^ (red) with irradiation and without irradiation. DMPO (50 mM); (**b**) UV–vis diffuse reflectance spectrum of samples; (**c**) XPS spectra of vacant BiCuSO and Pure BiCuSO. Left: Bi 4f spectrum. Right: O 2p spectrum.

Photocatalytic degradation of CR solution is associated with the formation of photo-degraded by active oxidation species. To further understand the above results, the EPR spin-trap technique (with DMPO) was carried out in the reaction process to determine the existence of the active species O_2_•^−^ and •OH. As illustrated in Figure 4a, there was no signal when the vacant BiCuSO suspension was in the dark. However, under light irradiation, four characteristic peaks with intensities of 1:2:2:1 for DMPO–•OH were observed in water suspension, indicating that the •OH radical was formed. Also, six characteristic peaks of DMPO–O_2_•^−^ can be observed in methanol dispersion under light irradiation. This indicates that O_2_•^−^ radicals are also generated on BiCuSO after light irradiation. Therefore, we conclude that the formation of O_2_•^−^ and •OH are responsible for the oxidation of the CR dye solution.

It is well-known that the defect should strongly affect the band structure of semiconductors. Thus, the UV–vis diffuse reflection spectrum was employed in order to study the optical absorption features of the samples. As shown in Figure 4b, both the pure BiCuSO and vacant BiCuSO have strong absorption in visible region (*k* ≥ 420 nm). However, the photocatalytic performance of vacant BiCuSO is much more enhanced compared with that of pure BiCuSO. UV–vis absorption spectra reveal that the band gap of the vacant BiCuSO is 1.5 eV, slightly smaller than the pure BiCuSO, which can be attributed to the triple vacant associates [25]. As we know, the bottom of the conduction band is located on the Bi atoms and the top of the valence band is located on the S and Cu atoms [26]. The vacant of Bi defects may induce a downward shift of the conduction band and cause band-gap narrowing which can better benefit the transition of electrons from valence band (VB) to conduction band (CB) enhancing the photocatalytic activity.

XPS surface elements analysis was performed to give a further understanding about the defect structure of BiCuSO. Compared with pure BiCuSO, the Bi 4f photoelectron peaks of vacant BiCuSO nanoplates shift to higher binding energy, indicating the formation of bismuth defects. In addition, as shown in Figure 4c, the binding energy of O 1s for vacant BiCuSO nanowires is about 530.5 eV, which is slightly smaller than that of the pure sample (531.5 eV). This shift may be attributed to weakness of the hybridization between Bi 6s and O 2p as a result of the introduction of surface bismuth defects [27]. Furthermore, the fitted O 1s (2) peak at lower energy is usually attributed to the oxygen atoms linked to the cations with lower electronegativity (such as O Bi) [25]. Compared with pure BiCuSO, it is found that the intensity of fitted O 1s (2) peak decreases with the formation of surface bismuth defects. This suggests that the average cation–oxygen bond (Bi–O) binding energy decreases as the triple vacant associates $V_{Bi}^{\prime \prime \prime }V_{O}^{••}V_{Bi}^{\prime \prime \prime }$ defects form, which not only enhanced the adsorption capability but also effectively separated the electron–hole pairs in the nanoplates.

The mechanism of the enhanced photocatalytic activity for the vacant BiCuSO is proposed as follows: BiCuSO has a unique layered structure of bismuth oxysulfide with the stacking of [Bi_2_O_2_]^2+^ layers and [Cu_2_Se_2_]^2−^ conductive layers along with z axis. This would induce the presence of internal static electric fields perpendicular to the [Bi_2_O_2_]^2+^ slab and [Cu_2_Se_2_]^2−^ slab, enabling the effective separation of the photoinduced electron−hole pairs. With the light irradiation, the self-induced internal electric fields could induce more efficient charge separation. In addition, the narrowing band-gap can also benefit the transition of electrons from valence band (VB) to conduction band (CB). Thus, the photoexcited electrons will be more easily transported to the surface of the nanosheets and be captured by the dissolved O_2_ to generate O_2_•^−^, which directly participate in the oxidation reaction, which can result in superior photocatalytic performance.

**Figure 5:**
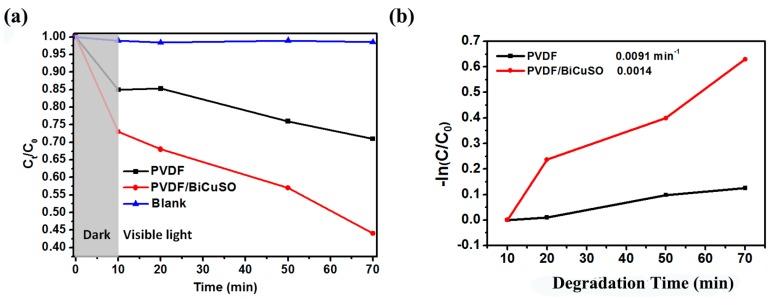
(**a**) Degradation rate of aqueous solution photocatalysis with PVDF and PVDF/BiCuSO as catalysts under visible light in 70 min. (**b**) Kinetic linear simulation curve of Congo Red photocatalytic degradation with as-prepared samples.

**Figure 6:**
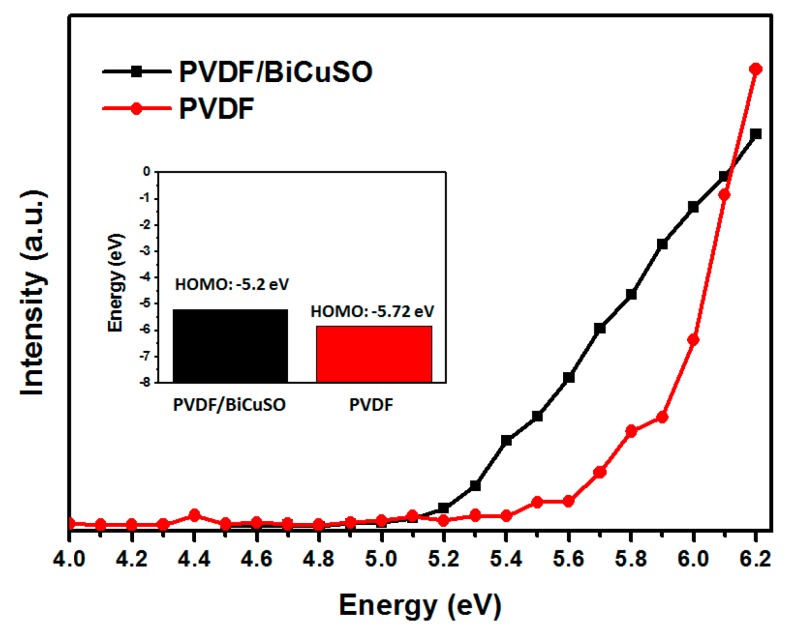
HOMO energy levels for PVDF and PVDF/BiCuSO.

Subsequently, to evaluate the photocatalytic performance of PVDF and PVDF/BiCuSO, photodegradation of CR under visible (*λ* > 420 nm) irradiation was investigated. As shown in Figure 5a, by loading the BiCuSO, the polymer matrix shows a fiercely improved photocatalytic activity. 55% of it was degraded in 70 min, twice as quickly as pure PVDF. However, an interesting phenomenon was found that both the PVDF and PVDF/BiCuSO shows promising performance in absorbing the organic pollutants, which confirms the potential application of the PVDF as a pollutant filter. To confirm the absorbing ability, the surface potentials of the PVDF and PVDF/BiCuSO were analyzed by photoelectron spectroscopy in air (PESA). As shown in Figure 6, compared with pure PVDF, the PVDF/BiCuSO composites show lower energy barrier for the electrons move to the surface of the nanofibers, confirming the effect of strong electronegativity of layered bismuth oxysulfide nanosheets on reinforcing the electric potential difference. To give a further investigation of the photocatalytic activity difference of the PVDF and PVDF/BiCuSO, the kinetic simulations with time (*t*) as abscissa and −ln(*C*/*C*_0_) as the vertical coordinate were fit and shown in Figure 5b. It is clear that the kinetic simulation curve is close to a linear curve, indicating the photocatalytic degradation follows pseudo first-order kinetics. The apparent reaction rate constant (*k*) is 0.0014 min^−1^ and 0.0091 min^−1^ for sample PVDF and vacant BiCuSO/PVDF, respectively. Significant enhancement of photocatalytic efficiency is demonstrated. The possible degradation mechanism for the nanocomposites may be as follows: The photocatalytic degradations of the dyes are influenced by the surface charge for the electrostatic interaction. The surface charge of metal oxide can be modified by F^−^. Studies show that F^−^ modified metal oxides show a better absorbability of the cationic dyes. Modified with PVDF, the contaminant will be more easily absorbed on the surface of the bismuth oxysulfide polymer nanocomposites (PVDF/BCuSO). Subsequently, under light irradiation, the photoexcited holes may be transferred rapidly due to the electrostatic attraction between the PVDF and the negatively charged [Bi_2_O_2_]^2+^ layers, and then participate in photocatalytic reaction. Therefore, the PVDF/BiCuSO nanofiber exhibits higher photocatalytic activity.

## 3. Materials and Methods

### 3.1. Preparation of Photocatalysts

Photocatalysts were fabricated by hydrothermal process as follows: 2.5 g Bi(NO_3_)_3_·5H_2_O (98%, Sinopharm), 1.3 g CuSO_4_ (98%, Sinopharm, Beijing, China), and 1 g thiourea (98%, Sinopharm, Beijing, China 99%) were dissolved in 60 mL NaOH solution (1 mol L^−1^) with the assistance of intense magnetic stirring. The suspension was ultrasonically treated for 30 min followed by subsequent vigorous magnetic stirring for another 30 min to get a highly uniform mixture. After that, the as-prepared precursor was sealed in a 100 mL Teflon-lined stainless autoclave (YZ-HR-100ML, Yanzheng, Beijing, China) and treated at 120 °C and 200 °C for 20 h respectively to get the vacant (B1) and pure BiCuSO (B2). The resulting black precipitates were segregated and washed with deionized water several times until the filtrate was neutral, and then it was dried at 80 °C for 12 h to obtain the final products.

### 3.2. Fabrication of PVDF@BiCuSO_Nanofibers via Electronspinning

PVDF@BiCuSO nanofibers were fabricated as follows: 0.3 g BiCuSO nanoplates were ultrasonically dispersed into 10 mL *N*,*N*-dimethylformamide (DMF) solution for 5 h. Then 1 g PVDF was added with vigorous magnetic stirring for 30 min. Then the mixture was stirred to form the precursor sol used for the following electrospinning process. The electrospinning was carried out in a syringe with applied electric field of 1.3 kV cm^−1^, resulting in a dense web of the electrospun composite nanofibers of PVDF@BiCuSO which were collected on the aluminum foil.

### 3.3. Characterization

Wide-angle X-ray powder diffraction (XRD) measurements were performed on X-ray diffractometer (Bruker, D8 Advance, Karlsruhe, Germany) using CuKa radiation (*k* = 1.5418 A). The morphologies were checked by field-emission scanning electron microscopy (FE-SEM, JSM-7001F, JEOL, Tokyo, Japan) and transmission electron microscopy (TEM, JEM-2100, JEOL, 200 kV, Tokyo, Japan). The UV–vis diffuse reflectance spectrum (DRS) was measured on UV–vis spectrophotometer (U3310, HITACHI, Tokyo, Japan) to investigate the optical properties. X-ray photoelectron spectroscopy (XPS) measurements were conducted on a Thermo XPS ESCALAB 250Xi instrument (Thermo Fisher, Waltham, MA, USA). The Brunauer–Emmett–Teller (BET) surface area was determined by Autosorb-iQ2-Mp (Quantachrome). Spin resonance signals of spin-trapped paramagnetic species with 5,5-dimethyl-l-pyrroline *N*-oxide (DMPO) were detected using the JES-FA200 EER spectrometer (JEOL, Akishima, Tokyo, Japan) with light irradiation. To measure the contact angle, the powders were pressed into pieces with diameters of 15 mm. Contact angles were measured on a contact angle system (Dataphysics, OCA15EC, San Jose, CA, USA) at ambient temperature. The average contact angle was obtained by measuring five different positions of the same sample.

The photocatalytic (powder) activity was characterized by degradation of Congo Red aqueous solution (110 mg/L) under the irradiation of visible light (*k* ≥ 420 nm, 650 mWcm^−2^) with 2 g/L photocatalyst. The photocatalytic (fibrous membranes) activity was characterized by degradation of Congo Red aqueous solution (11 mg/L) with 0.1 g/L photocatalyst. Typically, 0.16 g photocatalyst powder was dispersed into 80 mL CR solution, cooling-water bath and magnetic stirring were maintained continuously to prevent thermal effects during the degradation process and to maintain uniformity. A 300 W xenon lamp with 420 nm cut-off filters was used as visible light source. The incident light source was placed above the aqueous solution vertically with illumination intensity of 130 mW/cm^2^ at upper surface of the solution. At regular time intervals, 3 mL suspension was collected and centrifuged, and the residual CR concentration in the supernatant was analyzed by UV–vis spectrophotometer. The degradation rate was evaluated derived from the value of peak at 495 nm. HOMO energy levels were measured by photoelectron spectroscopy in air using a Riken Keiki AC-2 PESA spectrometer (Riken Keiki, Azusawa Itabashi-Ku, Tokyo, Japan), samples for PESA were prepared with a size 1 × 1 cm.

## 4. Conclusions

In summary, nanofiber membranes with excellent photocatalytic activity were successfully fabricated. The enhanced photocatalytic activity with the loading of ceramic nanoparticle was investigated. In addition to this, due to the high electronegativity of PVDF/BiCuSO fibers, synergistic effects of polymer matrix, and the BiCuSO, the nanofiber membrane shows an effect of absorbing organic contaminants. In particular, the $V_{Bi}^{\prime \prime \prime }V_{O}^{••}V_{Bi}^{\prime \prime \prime }$ will make the (101) facets negatively charged which will increase the electronegativity of the PVDF fiber and absorb more organic contaminant. In addition, the defects will enhanced the generation of hydroxyl radical and adsorption of cationic dye molecules onto the photocatalysts, which facilitates the photosensitization process. Our results provide some new insights into the excellent photocatalytic property of polymer nanocomposites and provide a new way for engineering the intrinsic defects on the surface of the loading nanoparticle for new types of nanofibrous materials, with applications in air purification or flexible optoelectronic devices.

## Figures and Tables

**Table 1:** The BET (Brunauer–Emmett–Teller) specific surface area of different samples.

Samples	Pure BiCuSO	Vacant BiCuSO
BET (m^2^/g)	26.40	31.64

**Table 2:** Specific surface area and reaction rate constant of as-prepared samples.

Samples	Pure BiCuSO	Vacant BiCuSO
K (10^−3^ m^−2^ min^−1^)	0.7	6.9
